# Large-Scale Lever-Based Triboelectric Nanogenerator for Sensing Lateral Vibration and Wrist or Finger Bending for Controlling Shooting Game

**DOI:** 10.3390/mi12091126

**Published:** 2021-09-18

**Authors:** Inkyum Kim, Tae Young Ahn, Daewon Kim

**Affiliations:** 1 Department of Electronics and Information Convergence Engineering, Kyung Hee University, 1732 Deogyeong-daero, Giheung-gu, Yongin 17104, Korea; inkyum.kim@khu.ac.kr; 2 Institute for Wearable Convergence Electronics, Kyung Hee University, 1732 Deogyeong-daero, Giheung-gu, Yongin 17104, Korea; 3 Department of Orthopaedic Surgery, Bio-Medical Research Institute, Pusan National University Hospital, 179 Gudeok-ro, Seo-gu, Busan 49241, Korea; 4 Department of Electronic Engineering, Kyung Hee University, 1732 Deogyeong-daero, Giheung-gu, Yongin 17104, Korea

**Keywords:** triboelectric nanogenerators, lever, vibration sensors, low detection limit, game controllers

## Abstract

With advances in internet of things technology and fossil fuel depletion, energy harvesting has emerged rapidly as a means of supplying small electronics with electricity. As a method of enhancing the electrical output of the triboelectric nanogenerator, specialized for harvesting mechanical energy, structural modification to amplify the input force is receiving attention due to the limited input energy level. In this research, a lever structure was employed for delivering the amplified input force to a triboelectric nanogenerator. With structural optimization of a 2.5 cm : 5 cm distance ratio of the first and second parts using two lever structures, the highest electrical outputs were achieved: a *V*_OC_ of 51.03 V, current density of 3.34 mA m^−2^, and power density of 73.5 mW m^−2^ at 12 MΩ in the second part. As applications of this triboelectric generator, a vertical vibration sensor and a wearable reloading trigger in a gun shooting game were demonstrated. The possibility for a wearable finger bending sensor with low-level input was checked using a minimized device. Enhanced low-detection limit with amplified input force from the structural advantage of this lever-based triboelectric nanogenerator device can expand its applicability to the mechanical trigger for wearable electronics.

## 1. Introduction

As the demand for electrical energy is continuously growing with the increasing number of wearable electronics and internet of things (IoT) devices, developing and using novel energy sources are recently attracting attention [1,2]. Additionally, the depletion of fossil fuels is accelerating the replacement of the fossil fuel-based conventional energy sources [3,4,5]. Therefore, multitudinous researchers have been focusing on how to harvest the ambient energy. A lot of energy is wasted in the form of heat, light, and sound [6]. In operating small electronics, the scavenged vibrational energy from humans, machines, and phenomena of nature can highly increase the operating time with the use of conventional batteries [7]. Accordingly, the energy harvesting technology can be an effective way to reuse the wasted energy.

In energy harvesting technology which captures and stores energy from external sources, solar cells (SCs), electromagnetic generators (EMGs), thermoelectric generators (ThEGs), piezoelectric nanogenerators (PENGs), and triboelectric nanogenerators (TENGs) are considered to be mainstream technologies. The SC harvests the light energy from the sun or other light sources with the principle of the photoelectric effect [8]. Direct current (DC) can be generated with this solar cell device mainly on sunny days or on cloudy days, even if its output would not be the best performance. The ThEG scavenges the thermal energy using the principle of the Seebeck effect [9,10]. Based on the temperature difference maintained between the top and bottom sides of the device, the ThEG can also generate stable DC output. EMGs, which consist of coils and magnets, have been widely used for generating electricity ever since Michael Faraday discovered the operating principle of the EMG in 1831 [11,12,13]. EMGs generate an alternate current by changing the magnetic flux in the coil, showing the high energy conversion efficiency in a large-scale device [14,15].

As mechanical energy harvesters, PENGs with using a piezoelectric material [16,17,18] and TENGs operating with a simple contact-separation motion [19] are widely used these days. Both of these mechanical energy harvesters have been used for generating electricity or sensing an external stimulus. These harvesters also present the advantages of light weight and possibility of miniaturization. In particular, the TENGs, which operate on the principle of the conjunction of contact electrification and electrostatic induction, show distinctive advantages in the easy material selection, simple structure, and high output power compared to other small energy harvesters [20,21]. From the easy material selection property, low-cost and eco-friendly materials can be adopted in parts of the TENG device. Moreover, the device can be easily fabricated without using complicated equipment.

To enhance the electrical output of the TENG without hybridizing, several methods were reported: material selection [22,23,24,25,26], contact surface modification [27,28,29,30,31], using an external circuit [32], and structural modification [33,34,35]. For example, a simple method to enhance the electrical output of the TENG is adopting two materials far from each other in the triboelectric series. Increasing the contact surface area of the dielectric or that of the counter material is another method for increasing the output in the first stage. Recently, some researchers have succeeded in generating high electrical output by using a power management integrated circuit (PMIC) as an auxiliary circuit [36,37,38]. Moreover, structural modification techniques were also adopted to effectively convert the limited input source into electrical energy.

In this paper, using the structural modification method, the electrical output of the TENG is increased with a simple machine structure of a lever. The lever consists of a bar to inject the force into the object and a fulcrum to transfer the injected force. When using the lever structure, higher output force can be applied to the object with smaller input force when the fulcrum is located closer to the object and farther from the injecting point at once. When this structure is applied to the TENG, the double electrical output from the first (hereafter ‘1st’) part (directly applied) and the second (hereafter ‘2nd’) part (beyond the fulcrum) of lever-based TENGs (L-TENGs) can be generated. With a commercial force sensor, the optimized point that can generate more output force can be found. The output from the 2nd part of the TENG can be increased with the optimized distance ratio of the fabricated device. Moreover, the fabricated L-TENG device can detect the small input force from the electrical output signals with the boosted output force at the 2nd part. Inductively coupled plasma (ICP) etching is applied to the contact-dielectric layers, further enhancing the electrical output of the L-TENG [39]. A finite element method (FEM) can be utilized to check the electrical potential distribution with variation of the distance ratio. Several electrical output characteristics of the frequency response, durability, and charging capacitors are analyzed to use this L-TENG as an energy harvester. LEDs are also illuminated by connecting and operating the L-TENG device to represent the ability for driving electronic devices. With additional structural advantages, this device can be applied to a vibration sensor and a wrist bending sensor with the enhanced accuracy from the output boosting characteristic. This L-TENG will open up a new path for enhancing energy harvesting efficiency and additional use of TENG devices as wearable sensors.

## 2. Materials and Methods

### 2.1. Fabrication of the L-TENG

The frames of the flat and ‘U’ shaped L-TENG devices were fabricated by three-dimensional (3D) printing. The filament was selected with the acrylonitrile butadiene styrene (ABS, ABS-A100) filament printed by a 3D printer (Cubicon 3DP-310F, Seongnam, Korea). The dimensions of the flat L-TENG device were checked with the values of the width of 19.7 cm, depth of 3.5 cm, and height of 4.6 cm. The ‘U’ shaped L-TENG device represented the dimensions of width of 13.5 cm, depth of 3.5 cm, and height of 8 cm. The size-decreased sample showed 46% of the initial device. The 2 mm-thick-acrylic plate with the attached films using double-sided tape was fixed to different locations on the printed frame with silicone hot melt glue. The electrode layer was constructed by attaching a piece of Al tape at the acrylic plate with the area of 3 × 2 cm^2^.

### 2.2. Surface Modification and Characterization of the PTFE Film

The ICP etching was implemented on the surface of the PTFE film to fabricate the micro-nano structure. The thickness, length, and width of the PTFE film were 200 µm, 2.5 cm, and 2 cm, respectively. The detailed etching process was conducted with flowing O_2_ plasma with a flow rate of 30 sccm and 30 W power was applied for five minutes. The scanning electron microscopy (SEM) image was taken with a high-resolution field emission-SEM (HR FE-SEM, Carl Zeiss MERLIN, Oberkochen, Germany).

### 2.3. FEM Analysis

COMSOL Multiphysics (COMSOL Inc., Stockholm, Sweden) was used to simulate the surface electric potential with a FEM. By changing the surface charge density and the distance between the top and bottom parts of TENGs, the electric potential difference between the two electrodes was analyzed with this program.

### 2.4. Measurement of the Electrical Output from L-TENG

An electrodynamic shaker (Labworks Inc. LW139.138-40, Costa Mesa, CA, USA) was used to apply input force to the 1st part-TENG. The shaker is controlled by connecting it to a function generator (Agilent Technologies, Inc. 33120A, Santa Clara, CA, USA). The input amplitude from the electrodynamic shaker was fixed at a point where the measured input force values at each 1st part- and 2nd part-TENGs with the same distance were the same. The electrical outputs of the open-circuit voltage (*V*_OC_) and short-circuit current (*I*_SC_) were measured by a system electrometer (Keithley Model 6514, Solon, OH, USA) through a DAQ system (NI PCI-6220, Austin, TX, USA). A force sensor (Dytran Instruments, Inc. 1053v4, Chatsworth, CA, USA) and an amplifier (Dytran Instruments, Inc. E4110C, Chatsworth, CA, USA) were used for checking the input force at the location of both the 1st part- and 2nd part-TENGs.

### 2.5. Setting up a Shooting Game Controller

For reload triggering of the gun in a shooting game, the voltage signal was transferred through the Arduino Uno board using the analog port. A serially connected load resistor of 1 GΩ was connected to both the 1st part- and 2nd part-TENGs. Unity (Unity Technologies, San Francisco, CA, USA) game engine was used to implement a gun shooting game, as shown in supplementary materials.

## 3. Results

### 3.1. Structure of the L-TENG Device and Surface Characterization of the Dielectric Layer

**Figure 1:**
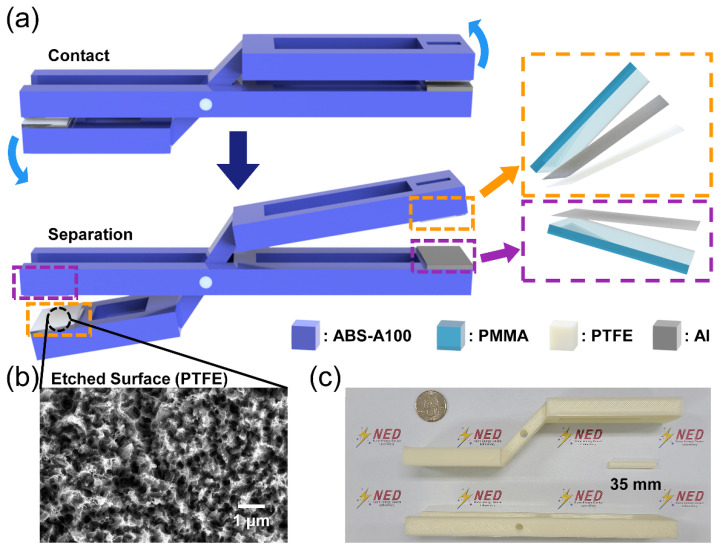
(**a**) Structural illustration of the lever-based TENG (L-TENG). (**b**) PTFE surface with ICP etching. (**c**) A digital camera image of the 3D printed flat L-TENG device.

The 3D printed structure and TENG parts of the L-TENG are depicted in Figure 1a. The white circle part of the structure combiner fixes both the axis and the fulcrum. The curved structure rotates in clockwise and counterclockwise directions, identical to the blue curved arrow, by an external force. The contact and separation states before and after rotating 3° are respectively displayed. Side views of each 3D printed part of the flat structure, curved structure, and structure combiner are displayed in Figure 1c with a quarter coin. The width, depth, and height of the L-TENG are 19.7 cm, 3.5 cm, and 4.6 cm, respectively. These dimensions of the L-TENG were adopted to allow the device to be put on the wrist and to measure electrical outputs with force-applying equipment due to the fixed size.

Both the left and right TENG parts are divided into two parts, as indicated by purple-colored and orange-colored boxes in Figure 1a. The purple-colored box illustrates the substrate and the outer electrode. A PMMA plate was used as a flat substrate and Al tape was attached to the substrate. The orange-colored box is similar to the purple-colored box, but a 200 µm-PTFE layer is additionally attached to the electrode layer as a dielectric layer of the TENG. These two outermost layers are in states of contact and separation with rotational motion of the printed structure.

The surface of the PTFE was etched through an ICP etching process with O_2_ gas. An SEM image of the etched surface is presented in Figure 1b. The morphology of the PTFE surface shows uniformly distributed nano-scale holes from tens to hundreds of nanometers. These holes can enhance the electrical output with this contact-separation mode TENG by increasing the contact surface. The results of the PTFE sample after going through the ICP etching process showed enhanced electrical outputs with 228% of the open-circuit voltage (*V*_OC_) and 193% of the short-circuit current (*I*_SC_), as presented in Figure S1.

### 3.2. Operating Mechanism of the L-TENG and FEM Analysis of the Electric Potential

**Figure 2:**
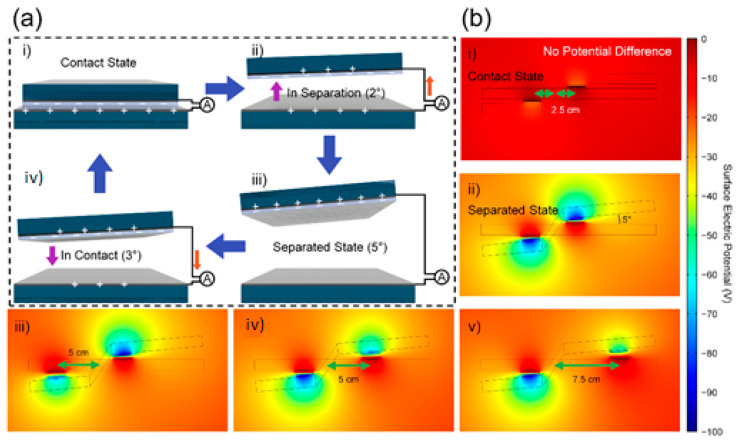
(**a**) Operation principle of the L-TENG. (**b**) FEM simulation results presenting the electric potential with different distance ratios.

The energy harvesting mechanism from the 1st part- and 2nd part-TENGs is depicted in Figure 2a. In Figure 2a(i), the contact state of the upper and lower layer is shown with a 0° displacement angle. In the contact state with the contact electrification, the PTFE layer and the Al layer become negatively and positively charged, respectively, according to the triboelectric series [40]. No current flows through the ammeter due to the stable state. When the two layers begin to separate, electrostatic induction occurs, and the positive charges partially flow from the bottom electrode to the top electrode in order to comply with charge neutrality in Figure 2a(ii). The current also flows from the bottom electrode to the top electrode. The number of positive charges at the top electrode and negative charges at the PTFE surface is counterpoised in the separation state in Figure 2a(iii). On the other hand, in Figure 2a(iv), the upper layer begins to rotate in the clockwise direction. The positive charges flow back to the bottom electrode and the current direction is opposite to that of Figure 2a(ii) with generation of alternative current (AC). One cycle comes to an end with the return to the first state and this cycle repeats with continuous counterclockwise and clockwise rotation through the external force.

The FEM was additionally used to show the surface electric potential profile. The dimensions were the same as the real size L-TENG. The distance from the fulcrum of 2.5, 5, and 7.5 and displacement angles of 0 and 5° between the upper and lower layers were changed as variables. The potential difference between the two electrodes was the same with the same distance of 2.5 cm regardless of the left-2nd part and right-1st part in Figure 2b. The potential differences at the distance of 2.5 cm was 23.9 V for contact and 93.5 V for separation in Figure 2b(i,ii). Therefore, the open-circuit voltage (*V*_OC_) between these two states with 2.5 cm-distance was 69.6 V. The *V*_OC_ presents a decreasing tendency with long distance due to the small contact force. More details can be elucidated with the following Equation (1):

*F*_1_*l*_1_ = *F*_2_*l*_2_, (1)

*F*_1_ and *F*_2_ are the forces at the 1st part- and 2nd part-TENGs. *l*_1_ and *l*_2_ are the distance between the fulcrum and each TENG part. It should be emphasized that the force can be increased with a shorter distance, and vice versa. The decreased input force can diminish the surface charge density of each contact layer and *V*_OC_ also displays the same tendency of decreasing. The *V*_OC_ from the distance of 5 cm showed a value of 50.2 V regardless of the 1st part- and 2nd part-TENGs in Figure 2b(iii,iv). In the case of the distance of 7.5 cm in the 1st part in Figure 2b(v), the *V*_OC_ was further decreased to 33.2 V. These simulation results will be experimentally examined in the following section.

### 3.3. Input Force at L-TENG and Electrical Output Parameters by Changing the Gap Distance and Distance Ratio

The input force to the 1st part- and 2nd part-TENGs, respectively, was measured with the ‘U’ shaped structure to draw more accurate results due to the existence of a prop at the 2nd part-TENG. Compared with the flat structure in Figure S2a,b, the ‘U’ shaped structure can be fixed at a salient due to its structural property as depicted in Figure S2c,d. Therefore, for measuring the applied force with changing the distance ratio, using the ‘U’ shaped structure showed an advantage for excluding the effect of the gravitational force. Figure S2c,d represent the contact and separation states, respectively. The fixture held the inner structure and the outer structure vibrated with connection to the shaker. Force can be sensed with contact motion of the force sensor and an acrylic plate. This plate played the role of correcting the height to constitute the same condition as the contact of the two triboelectric layers. Digital camera images of the outer structure containing the force sensor are displayed in Figure S3a,b. The force sensor can be located at both the top and bottom part of this outer structure. The inner structure is also illustrated in Figure S3c. These two structures were assembled with use of the same structure combiner in Figure 1c.

**Figure 3:**
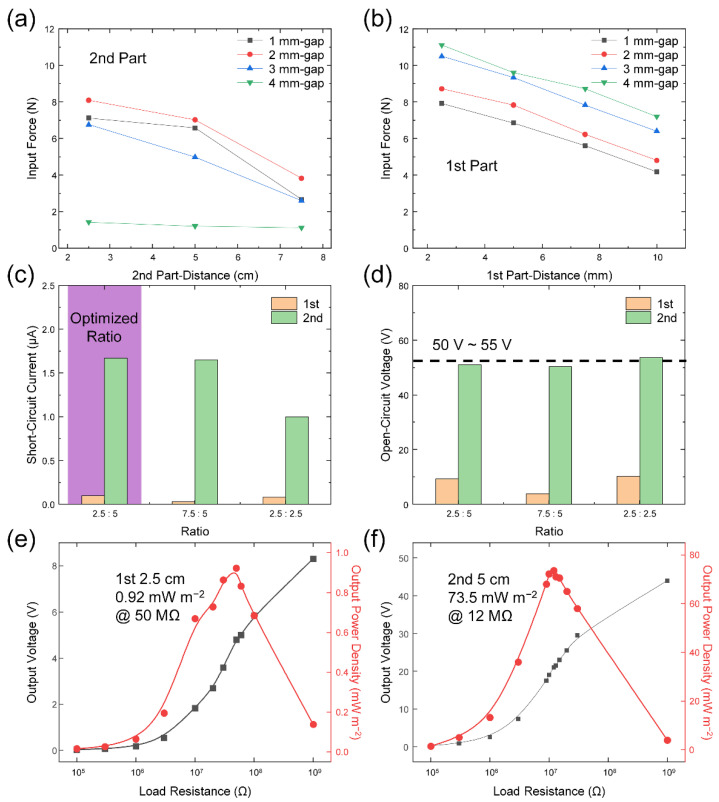
Input force and basic electrical characteristics of the fabricated L-TENG. Input force of the (**a**) 2nd part- and (**b**) 1st part-TENGs with different gap-distance between two layers of the 2nd part. (**c**) *I*_SC_ and (**d**) *V*_OC_ of the different distance ratio. Output power density with the (**e**) 1st part- and (**f**) 2nd part-TENGs.

The input force by the contact-separation motion was checked with varying gap distance between the flat and curved structures at the 2nd part. The 1st part was kept at the same distance of 4 mm to enhance the output at the 2nd part, which is the main part of the L-TENG device. From the point of view of distance from the fulcrum to TENG, the input force was weakened with long distance at the 2nd and 1st parts in Figure 3a,b, respectively. This result is consistent with Equation (1). Due to the size limitation of the force sensor with 1.9 cm-diameter, the gap cannot be shorter than 2.5 cm.

For structural optimization, the input forces with different gap distances in the 2nd part are compared in Figure 3a. The 4 mm gap showed the smallest values due to the contact at the 1st part and as a result of consuming most of the input force. The input force gradually strengthened with the smaller gap distance and the peak was shown at 2 mm distance. After the gap distance of 2 mm, the input force decreased since the force sensor could not readily achieve the separation state. Therefore, the gap distance of 2 mm was selected as an optimized structure and used in the study presented in the following section. The input force at the 1st part showed a gradual decreasing tendency with a greater gap due to the divided input force to the 2nd part in Figure 3b.

After adopting the L-TENG with the gap distance of 2 mm, electrical outputs from this device were measured with variation of the distance ratio using the flat structure in Figure 1a. The displacement of the shaker head was experimentally modified to apply the highest input force to the L-TENG at the 2nd part and fixed to a range. By fixing the distance from the fulcrum to TENG at the 2nd part, the *V*_OC_ and *I*_SC_ were measured while changing the 1st-distance from 2.5 cm to 7.5 cm in Figure 3c,d, respectively. In this comparison, both the *V*_OC_ and *I*_SC_ at the 2.5 cm-distance showed the highest outputs from the 1st part-TENG, which are identical results to the input force of the red line in Figure 3b. Therefore, the distance of the 1st part-TENG was fixed at 2.5 cm, presenting a *V*_OC_ of 9.33 V and an *I*_SC_ of 99.5 nA, which are about three-fold greater than the 7.5 cm case. The *V*_OC_ and *I*_SC_ from the 2nd part-TENG were the same when changing the distance from 2.5 cm to 7.5 cm due to the same input force.

By changing the distance in the 2nd part from 2.5 cm to 5 cm, the electrical outputs were measured as well. The *V*_OC_ slightly increased by 5% from 51.03 V to 53.62 V upon decreasing the distance due to the increased input force, indicated by the dotted line in Figure 3d. However, the *I*_SC_ decreased by 40% from 1.67 µA to 1.00 µA through the decreased distance from the shorter separation distance. This shorter separation distance of the 2.5 cm case than in the 5 cm case can cause the *I*_SC_ to decrease with low velocity in the separation process. The following Equation (2) represents the relationship between the *I*_SC_ and the separation velocity of the contact mode TENG [41]:

*I*_SC_ = *Sσd*_0_*v*(*t*)/(*d*_0_ + *x*(*t*))^2^, (2)

*S* and *σ* are the contact area of the metals and the tribo-charge surface density, respectively. *d*_0_ represents the effective thickness of the dielectric layer, which is divided by the dielectric constant of the material. *x*(*t*) and *v*(*t*) correspond to the distance between the two contact layers and the velocity of contact and separation of the two contact layers, respectively. Thus, 2.5 cm : 5 cm was selected as the optimal distance ratio for the 1st part- and 2nd part-TENGs, emphasized by the purple region in Figure 3c. The raw data and waveforms of *V*_OC_ and *I*_SC_ of this 2.5 cm : 5 cm case are represented in Figure S4.

The output power density was measured with each part of L-TENG in Figure 3e,f. The output power can be calculated using the following Equation (3):

*P* = *V*^2^/*R*, (3)

*P*, *V*, and *R* represent the output power, peak-to-peak output voltage, and load resistance, respectively. The output power density can be obtained by dividing the output power into the contact area of 5 cm^2^. The 1st part-TENG with 2.5 cm distance displayed the maximum output of 0.92 mW m^−2^ at 50 MΩ in Figure 3e and the 2nd part-TENG with 5 cm distance exhibited a maximum output of 73.5 mW m^−2^ at 12 MΩ in Figure 3f. These output power results showed a more definite difference comparing the outputs of the 1st part- and 2nd part-TENGs than in the *V*_OC_ and *I*_SC_. Moreover, the output powers of the 2.5 cm : 2.5 cm case are illustrated in Figure S5. The 1st part- and 2nd part-TENGs showed output power densities of 1.34 mW m^−2^ at 50 MΩ and 25.0 mW m^−2^ at 20 MΩ. Compared with the 2.5 cm : 5 cm case, the output power of the 1st part-TENG slightly increased due to the higher applied force at the 1st part. On the other hand, the 2nd part-TENG displayed three times lower output power from the dramatically decreased *I*_SC_ through the decline in the separation distance. The reason for using the optimized device of the 2.5 cm : 5 cm case was demonstrated once again.

### 3.4. Frequency Response, Durability Test, and Additional Electrical Output Performance of the L-TENG

**Figure 4:**
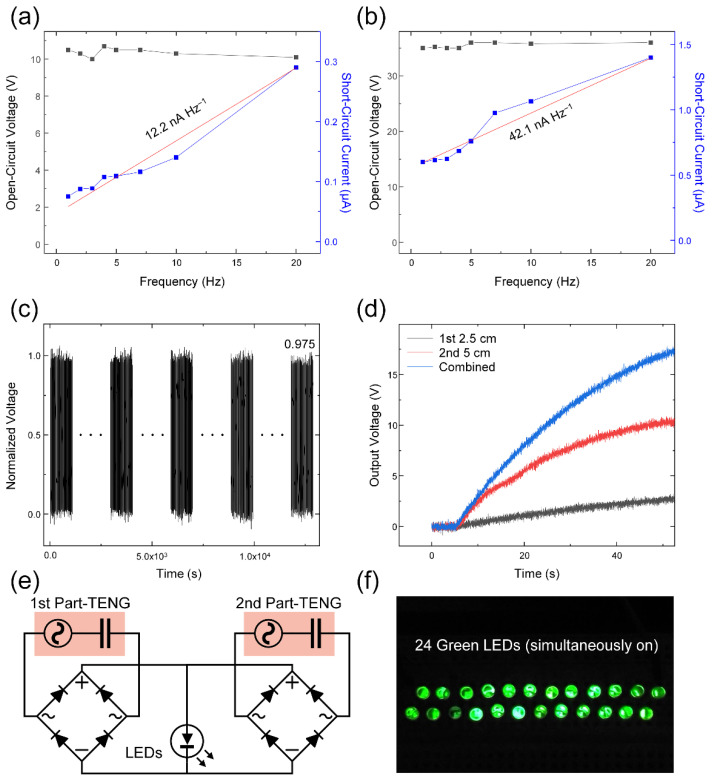
Additional electrical output characteristics of the fabricated L-TENG. Frequency response with the (**a**) 1st part- and (**b**) 2nd part-TENGs. (**c**) Durability test of the 2nd part-TENG with 2 Hz-input. (**d**) Charging ability to 0.1 µF capacitor with single and both L-TENG devices. (**e**) The circuit diagram for illuminating LEDs with the 1st part- and 2nd part-TENGs. (**f**) Illumination of the 24 green LEDs with the circuit composition of (**e**).

Additional electrical characteristics of the frequency response and durability were checked with the optimized L-TENG device. The electrical outputs from the 1st part-TENG and 2nd part-TENG are illustrated in Figure 4a,b, respectively. Both *V*_OC_s remained constant due to the fixed contact force. However, the *I*_SC_s were proportional to the contact-separation speed according to Equation (1). In Figure 4a, the *I*_SC_ of the 1st part-TENG increased from 0.08 µA (at 1 Hz) to 0.29 µA (at 20 Hz) with a gradient of 12.2 nA Hz^−1^, but the *V*_OC_ presented a small range of values from 10 V to 10.7 V. In accordance with the results of *V*_OC_ and *I*_SC_ in Figure 3c,d, the 2nd part-TENG displayed higher output values than in the 1st part-TENG. Values of 35 V to 36 V of the *V*_OC_ and 0.6 µA to 1.4 µA of the *I*_SC_ with a gradient of 42.1 nA Hz^−1^ were obtained by conducting this measurement in Figure 4b. When the different frequency of input signals was injected, the frequency can be observed by double accuracy with two outputs of the 1st part- and 2nd part-TENGs.

To use this L-TENG for a vibration sensor as well as an energy harvester, the durability should be checked with long time operation. With the 2 Hz input vibration to both the 1st part- and 2nd part-TENGs, the durability of the L-TENG was checked in 25,200 cycles during 12,600 s in Figure 4c. The output voltage decreased by only 2.5% after this continuous contact and separation-motion.

Moreover, the output characteristic of the L-TENG through a charging capacitor of 0.1 µF was studied with 2 Hz input. As with the relation of the *V*_OC_ and *I*_SC_ from the 1st part- and 2nd part-TENGs in Figure 3c,d, the 2nd part-TENG shows a faster and greater charging characteristic of 10.3 V than in the 1st part-TENG of 2.7 V in Figure 4d. The combined L-TENG with the parallelly connected and rectified 1st part- and 2nd part-TENGs provided the highest output voltage, 17.3 V. Configuring the circuit as in Figure 4e, a test for illuminating LEDs with the combined and rectified L-TENG was conducted and 24 commercial green LEDs were simultaneously operated in real time without a charging process, as seen in Figure 4f and Video S1. From these tests of the charging capacitor and illuminating LEDs, it can be suggested that the L-TENG can be used as an energy harvester for operating small electronics.

### 3.5. Applications of the L-TENG

**Figure 5:**
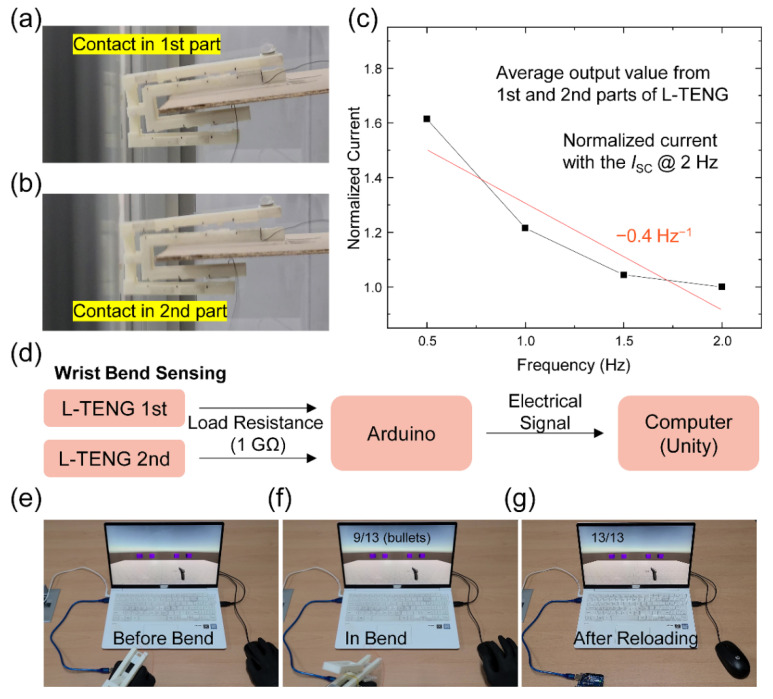
Applications of the proposed L-TENG. (**a**,**b**) Digital camera image of the setup for sensing vibration. (**c**) Electrical output current result of the vibration sensing. (**d**) Flowchart for sensing wrist bending and triggering reloading motion. Captured image of the gun shooting game: (**e**) after shooting four bullets, (**f**) in bending wrist, and (**g**) after reloading.

Two practical applications of the L-TENG are displayed in Figure 5. To enhance the scalability of the L-TENG in vertical movement, the 1st part- and 2nd part-TENGs respectively consisted of a single electrode TENG without an electrode under the PTFE layer, in contrast to the double-electrode TENG used in the previous experiments. The first application was constructed to measure the vibration frequency applied to a plywood plate. The ‘U’ shaped structure which was used to measure the input force in Figure 3a,b was adopted to effectively harvest and sense a vibrational signal with the curved structure. The contact states of the 1st part- and 2nd part-TENGs are captured in Figure 5a,b, respectively, and the continuous movement of the L-TENG can be checked in Video S2. The average peak values of the 1st part- and 2nd part-TENGs, which were normalized with the value at 2 Hz, are indicated in Figure 5c and show a decreasing tendency with a gradient of −0.4 Hz^−1^. With rising input frequency, the magnitude of vertical vibration decreased due to the destructive interference by the residual vibration from the past input shown in Video S2.

As the second application, a wearable controller for a gun shooting game was designed. The connection for operating this controller is illustrated in Figure 5d. Both the 1st part- and 2nd part-TENGs were connected to two analog channels of an Arduino board with a serially connected 1GΩ-load resistors to obtain a voltage signal from the L-TENG that can be distinguished from the noise. This electrical signal at the Arduino board was transmitted to the computer and operated as a trigger in the Unity program. The motion of wrist bending and contact separation states are displayed in Figure S6. After shooting the gun in the game with a left click of the computer mouse, the bullet will run out, as shown in Figure 5e. To reload the bullets, both output voltages from the 1st part- and 2nd part-TENGs needed to exceed the threshold point with the wrist bending in Figure 5f. By double checking the output electrical signal from the 1st part- and 2nd part-TENGs, the accuracy of the trigger in the game can be enhanced compared with the use of only one TENG device. When the trigger succeeded, the bullets filled the magazine, as seen in Figure 5g. The continuous operating process of this gun shooting game with the trigger is shown in Video S3. The program setting in Unity and other conditions for playing the game are shown in Figure S7. From these two applications of the vibration sensing and shooting game-controller, the electrical outputs from L-TENG can be used in the real world with large scalability to the diverse fields of wearable devices and sensors with double accuracy.

**Figure 6:**
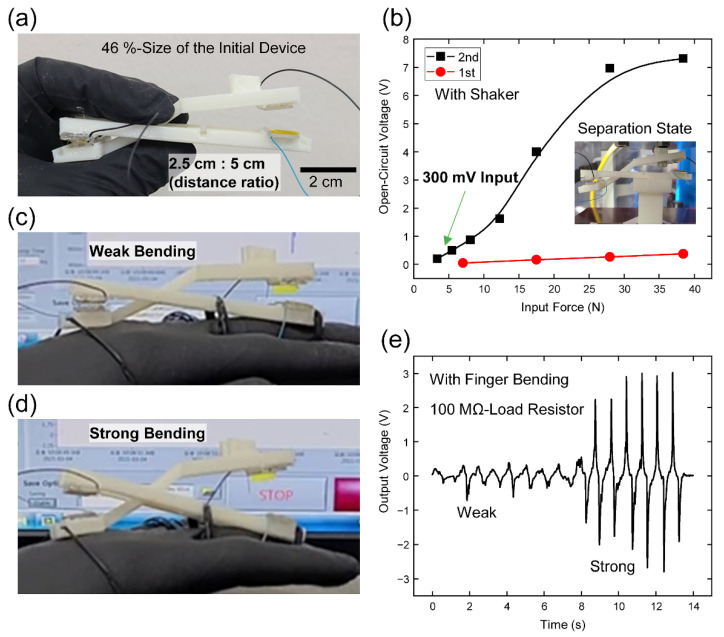
Weak-bend sensing ability of the L-TENG. (**a**) Digital camera image of the L-TENG with decreased size. (**b**) *V*_OC_-force characteristic with the 1st part- and 2nd part-TENGs. Digital camera images for displaying the finger bending and operating states of L-TENG with (**c**) weak bending case and (**d**) strong bending case. (**e**) Output voltage results of L-TENG from the two finger bending states.

As another application, a finger bending sensor was realized with a minimized device showing the 46%-length of the initial size in Figure 6a. To maximize the effect of amplifying the input force with the lever structure, the 1st part-TENG was designed to be in a non-contact state. The distance ratio was fixed to the optimized value of 2.5 cm : 5 cm corresponding to the 1st part- and 2nd part-TENGs, respectively. The experimental setup for measuring this force response of the minimized device is displayed in the inset of Figure 6b. In the measurement of *V*_OC_ and *I*_SC_ with changing the input force from 3.4 N to 38.4 N and the constant input frequency of 1 Hz, the *V*_OC_ and *I*_SC_ from the 2nd part-TENG in the minimized device were represented the increasing trend in Figure 6b and Figure S8, respectively. Due to the weak contact state, the electrical outputs from the 1st part-TENG showed relatively smaller values compared with that from the 2nd part in Figure 6b and Figure S8. Although the output levels were significantly low, they also displayed slight increasing trends with the increasing force over 7 N from the farther separation distance. It is worth noting that the outputs from the 2nd part-TENG were detectable with the force of 3.4 N and short separation distance under 1 mm by using the lever structure.

This minimized low-detection limit property of the L-TENG device can be applied for detecting the small finger bending. The minimized L-TENG was fixed at the outside of the hand for responding to the movements in the third metacarpophalangeal (MCP) joint. The photographs of the separation states of the L-TENG with the weak and strong bending states were displayed in Figure 6c,d and Video S4. The load resistor with 100 MΩ was used to prevent the floating signal and adjust the zero-cross point. Even though the electrical output level was decreased with the existence of the load resistance, the distinguishable peaks were shown in this test with the values of 0.3 V and 2.7 V at the weak and strong bending conditions, respectively in Figure 6e. The size of this L-TENG can be further decreased and that device can be attached to other body parts with generating small movements. Additional physiological data will also be collected with using this L-TENG device.

## 4. Discussion

In this study, a lever-based TENG was fabricated by simple 3D printing technology and analyzed with force and electrical outputs. The two double-electrode TENGs were adopted in the 1st part- and 2nd part-TENGs and ICP etching was conducted on the dielectric layer of the PTFE film. The FEM simulation presented higher electric potential in a shorter distance between the axis and the TENG due to the stronger contact force. The compressing force of the lever structure shows a higher value inversely proportional to the distance from the fulcrum using the ‘U’ shaped L-TENG device. With the use of the force sensor, the input force to each part of the TENG was measured with a 2 mm-gap of the 2nd part-TENG. The 2 mm gap case showed the highest input force in the 2nd part due to the effective separation and transmitted force. Moreover, the flat L-TENG device with the distance ratio of 2.5 cm of the 1st part and 5 cm of the 2nd part displayed optimal electrical outputs of the *V*_OC_ of 51.03 V and a current density of 3.34 mA m^−2^ in the 2nd part-TENG and a *V*_OC_ of 9.33 V and a current density of 0.199 mA m^−2^ in the 1st part-TENG. Each part of the TENG presented power density of 0.92 mW m^−2^ at 50 MΩ and 73.5 mW m^−2^ at 12 MΩ in the 1st part- and 2nd part-TENGs, respectively. With this condition, the 1st part- and 2nd part-TENGs showed the frequency response with gradients of 12.2 nA Hz^−1^ and 42.1 nA Hz^−1^, respectively. The durability of the 2nd part-TENG was verified with 2.5% degradation after being measured for 25,200 cycles. Combining the electrical outputs from the 1st part- and 2nd part-TENGs, the output voltage through the charging capacitor increased compared with the separated case and it can turn on 24 green LEDs. As practical applications, a vertical vibration sensor with a sensitivity of −0.4 Hz^−1^ and a wrist bending sensor trigger in a gun shooting game were designed with the use of the L-TENG. As a finger bending sensor, the minimized L-TENG showed the considerably low-detection limit of input force and the ability to distinguish between the weak and strong bending conditions. This L-TENG shows potential for enhancing the output from the lever structure and can be applied to a wearable sensor with the enlarged detection range from the structural advantage of the lever.

## Supplementary Materials

The following are available online at https://www.mdpi.com/article/10.3390/mi12091126/s1, Figure S1: Electrical outputs representing the effect of etching on PTFE surface, Figure S2: Schematics for flat and ‘U’ shaped L-TENG devices, Figure S3: Digital camera images of ‘U’ shaped L-TENG, Figure S4: Raw data of electrical outputs with the 2.5 cm : 5 cm distance ratio, Figure S5: Output power density with the 2.5 cm : 2.5 cm distance ratio, Figure S6: Digital camera images for before and after wrist bending, Figure S7: Unity program images for the gun shooting game, Figure S8: Current-force curve with the small L-TENG, Video S1: Video of illuminating LEDs with L-TENG, Video S2: Video of vibration sensing test with L-TENG, Video S3: Video of reloading trigger with L-TENG in the gun shooting game, Video S4: Video of detecting finger bending intensity with the small L-TENG.
