# Supplementary material for: Large-Scale Lever-Based Triboelectric Nanogenerator for Sensing Lateral Vibration and Wrist or Finger Bending for Controlling Shooting Game
Source: Micromachines (Basel). 2021 Sep 18;12(9):1126. doi: 10.3390/mi12091126 (PMC8466037; doi:10.3390/mi12091126)
Supplement: Supplementary file 1 [file micromachines-12-01126-s001.zip › micromachines-1342274-supplementary materials.pdf]

micromachines

Supporting Information

Large-Scale Lever-Based Triboelectric Nanogenerator for Sensing Lateral Vibration and Wrist or Finger Bending for Controlling Shooting Game

Inkyum Kim [1,2], Tae Young Ahn [3] and Daewon Kim

1. Electrical outputs representing the effect of etching on PTFE surface

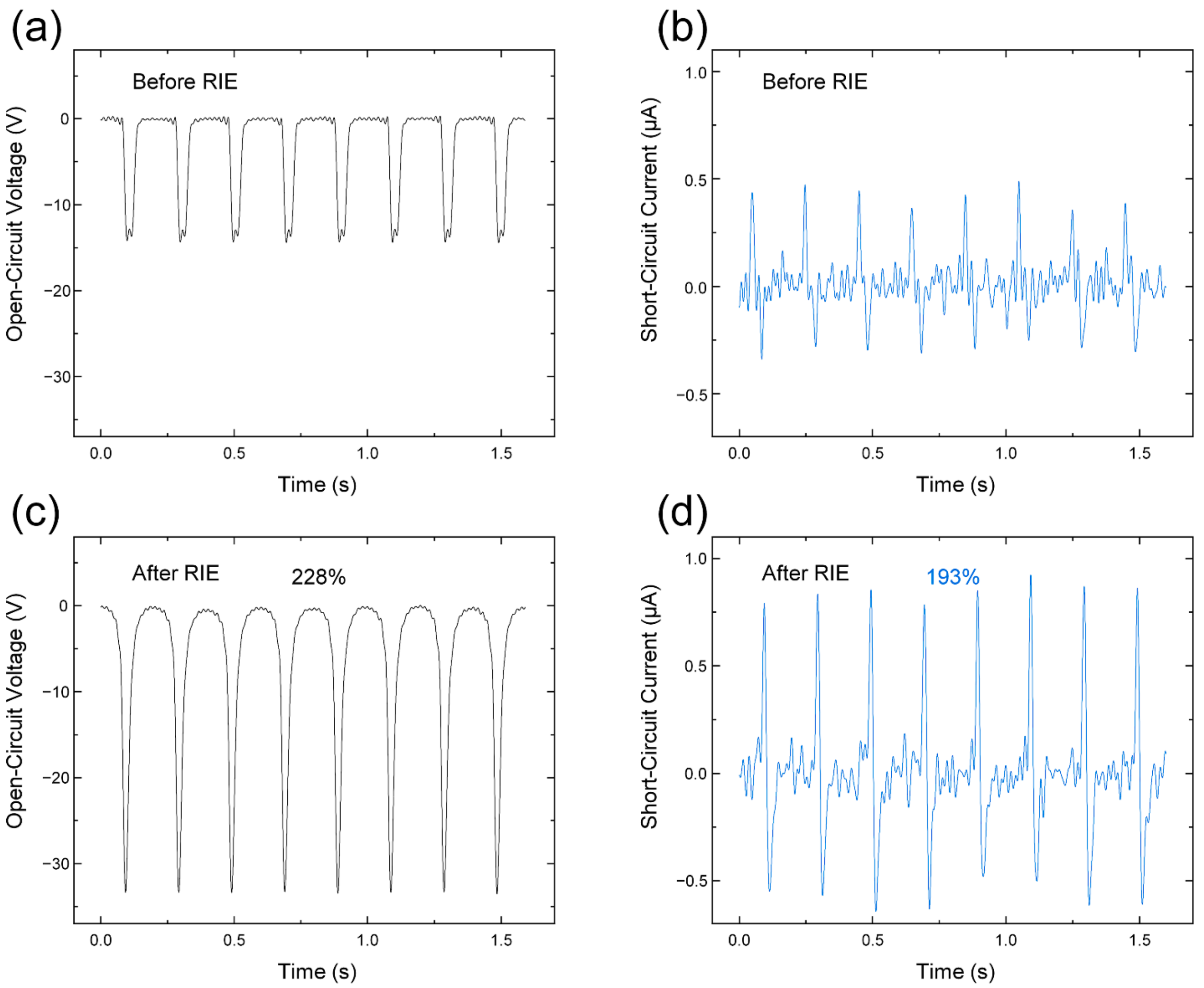

Figure S1. V_{OC}s and I_{SC}s of the double electrode TENG. Electrical outputs (a, b) before etching and (c, d) after etching the PTFE film.

2. Schematics for flat and 'U' shaped L-TENG devices

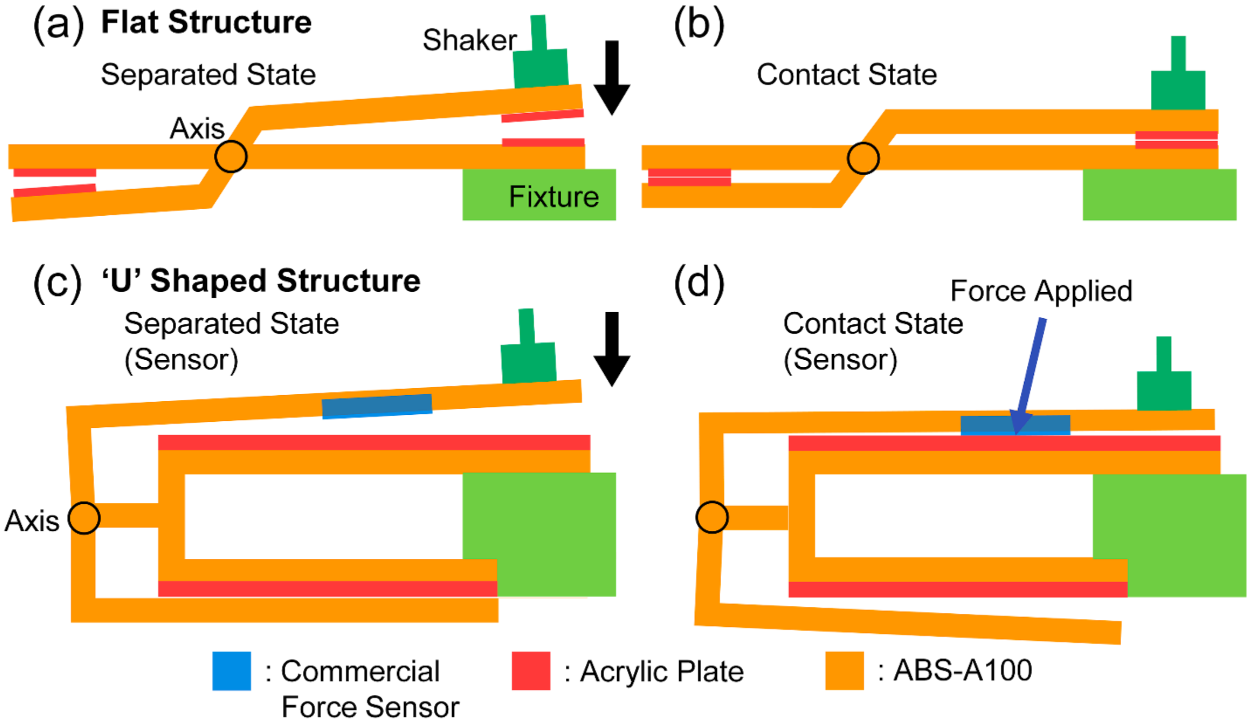

Figure S2. Schematic diagram for the two structures of the L-TENG. (a) Separation state and (b) contact state of the flat L-TENG device. (c) Separation state and (d) contact state at top 1st part of the 'U' shaped L-TENG device.

3. Digital camera images of 'U' shaped L-TENG

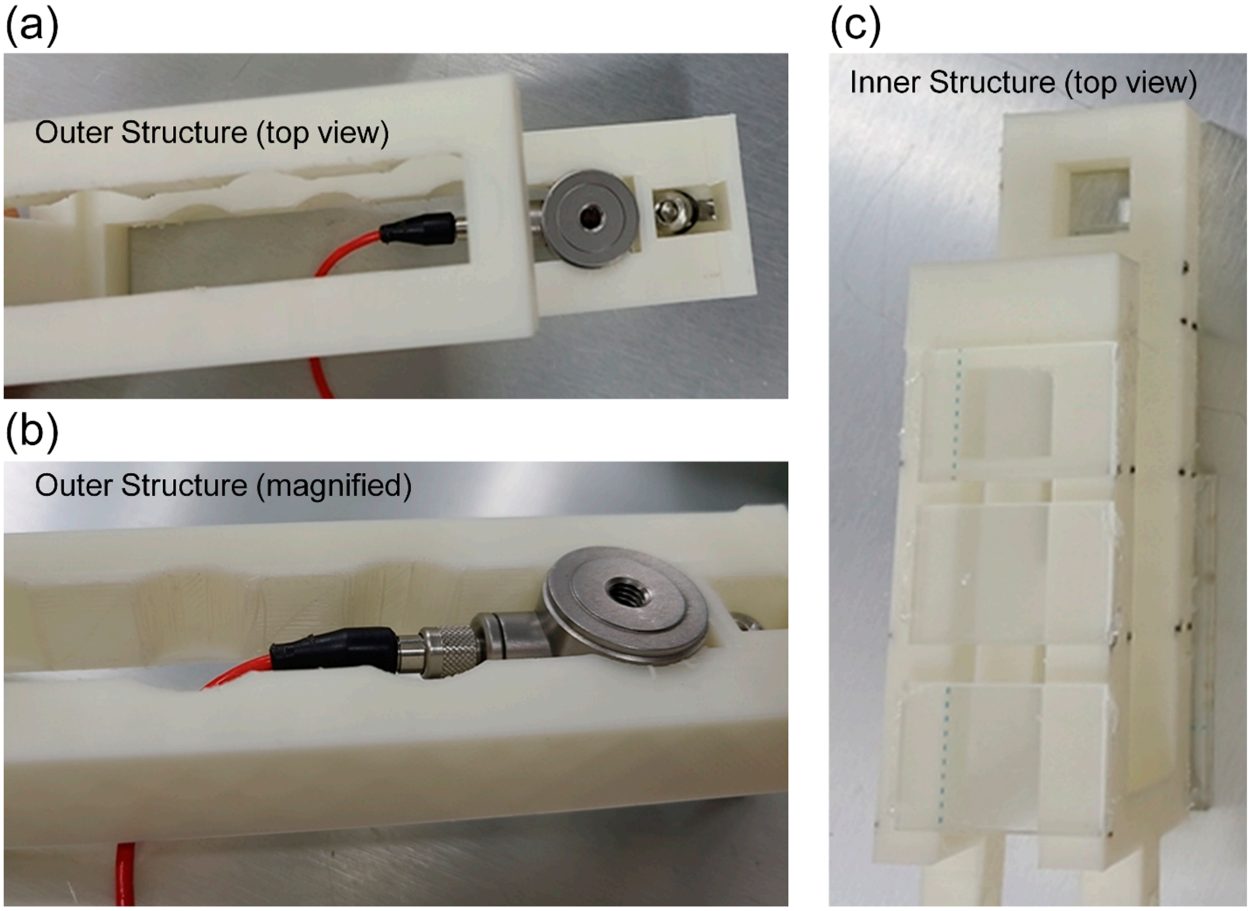

Figure S3. Digital camera images for the 3D printed structure for sensing the force input at top view of (a) outer structure part, (b) magnified part with force sensor, and (c) Inner structure part.

4. Raw data of electrical outputs with the 2.5 cm : 5 cm distance ratio

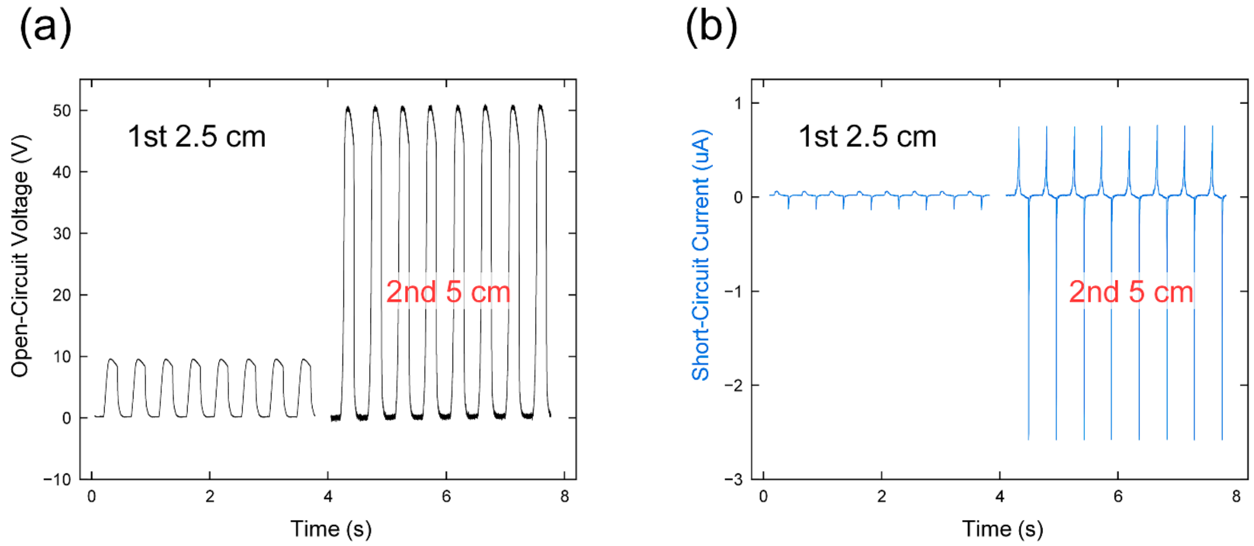

Figure S4. (a) V_{OC} and (b) I_{SC} of the 1st part-TENG with 2.5 cm and 2nd part-TENG with 5 cm.

5. Output power density with the 2.5 cm : 2.5 cm distance ratio

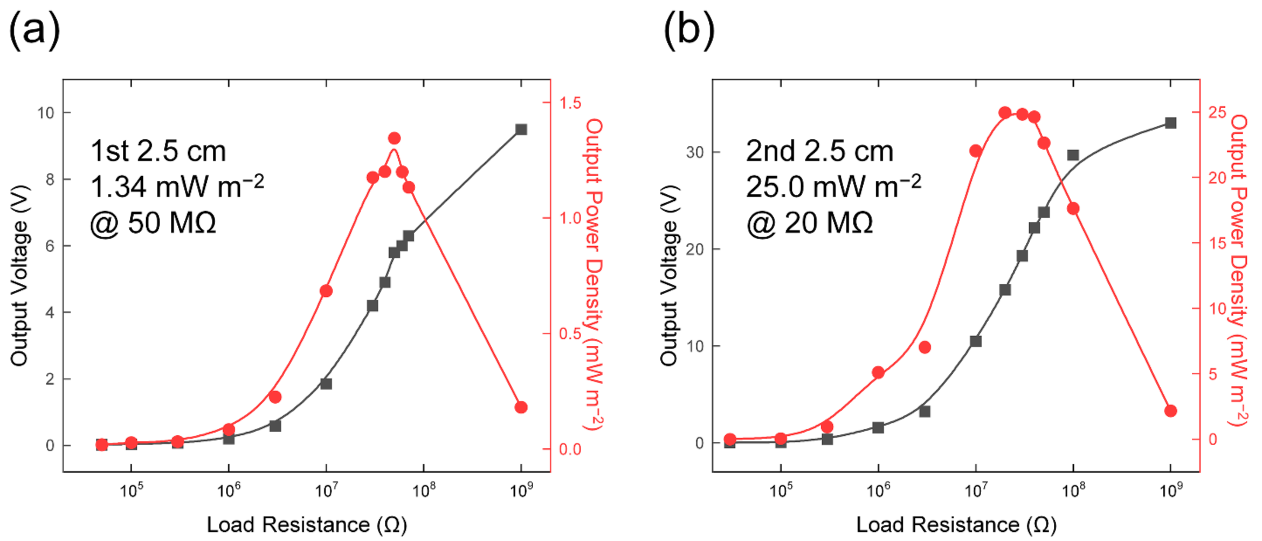

Figure S5. Output power density of the (a) 1st part-TENG with 2.5 cm-distance and (b) 2nd part-TENG with 2.5 cm-distance.

6. Digital camera images for before and after wrist bending

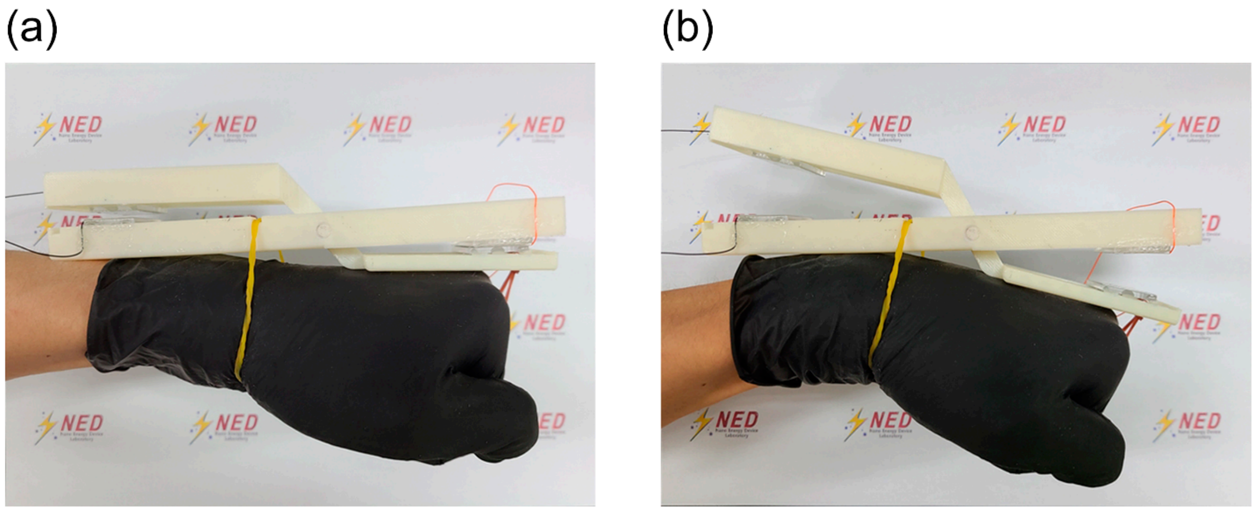

Figure S6. Digital camera images of (a) before bending and (b) after bending wrist.

7. Unity program images for the gun shooting game

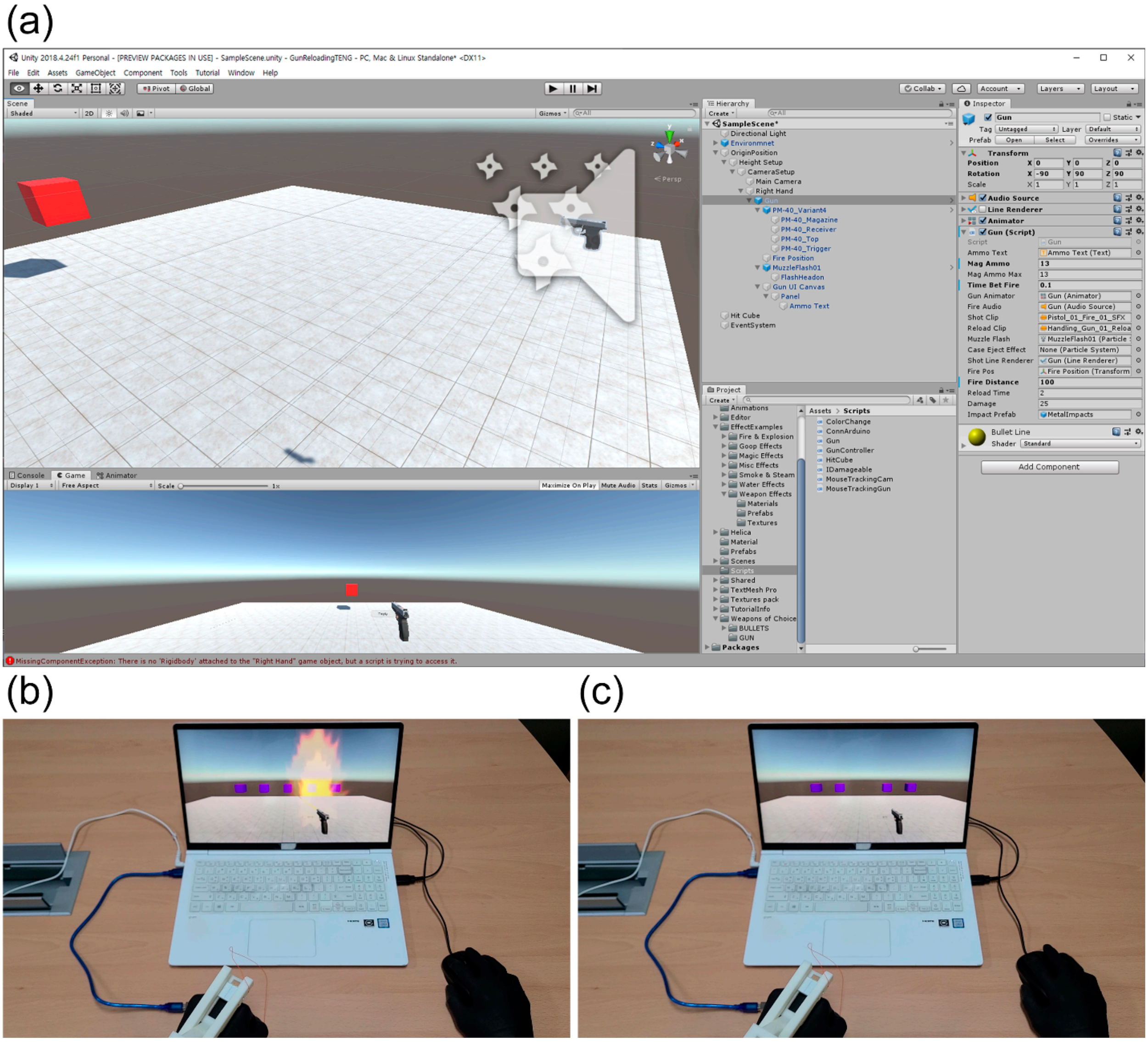

Figure S7. (a) Unity program screenshot for gun shooting game with fabricated objects. Captured images with gun shooting game (b) in shooting and (c) after shooting 4 bullets.

8. Current-force curve with the small L-TENG

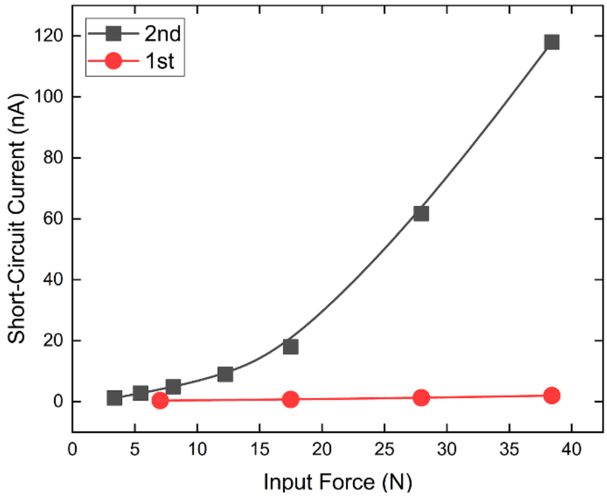

Figure S8. Output current from injecting the weak forces to the small L-TENG device.

9. Video of illuminating LEDs with L-TENG

Video S1. Simultaneously illuminating 24 serially connected green LEDs using a rectifier with 1st part- and 2nd part-TENGs.

10. Video of vibration sensing test with L-TENG

Video S2. Vibration sensing test displaying the state of L-TENG with 4 different frequency of 0.5, 1, 1.5, and 2 Hz of the input.

11. Video of reloading trigger with L-TENG in the gun shooting game

Video S3. The gun shooting game with aiming, shooting with mouse, and reloading with L-TENG.

12. Video of detecting finger bending intensity with the small L-TENG

Video S4. Detection of both the weak and strong finger bending using the small L-TENG device.
